# Effect of adult day care centre attendance on hypertension management

**DOI:** 10.4102/safp.v66i1.5961

**Published:** 2024-08-08

**Authors:** Sentebaleng E. Seitshiro, Omololu S. Aluko, Wilhelm J. Steinberg

**Affiliations:** 1 Department of Family Medicine, Faculty of Health Sciences, University of the Free State, Bloemfontein, South Africa; 2 Department of Biostatistics, Faculty of Health Sciences, University of the Free State, Bloemfontein, South Africa

**Keywords:** adult day care centre, attendance, hypertension, adherence, treatment

## Abstract

**Background:**

Hypertension (HT) silently threatens one in three adults, especially older adults, who struggle with blood pressure (BP) control because of limited health access, poor adherence to medication and failure to make lifestyle changes. This increases their risk for heart disease, kidney failure and dementia. Fortunately, adult day care centres (ADCCs) offer hope. These community facilities provide daytime care, including health support services, social activities and exercise. This study investigated the perceived effect of adult daycare centre attendance on BP control and treatment adherence.

**Methods:**

A mainly descriptive study at MUCPP Community Health Centre (CHC) in Bloemfontein, South Africa, between July 2020 and September 2020, used questionnaires researchers completed during interviews. The researchers approached 372 hypertensive patients of a minimum of 60 years old and at least 2 years since being diagnosed.

**Results:**

Of the 90 who attended ADCCs, 71.1% had controlled hypertension compared to 51.4% of those who did not. While treatment adherence showed no difference, a positive association between ADCC attendance and BP control is evident.

**Conclusion:**

The findings suggest a promising link between ADCC attendance and improved BP control in older adults with hypertension. Adult Day Care Centres warrant further exploration as it seems to be an encouraging support intervention for this vulnerable population.

**Contribution:**

This study highlights the positive impact ADCCs have on hypertension management in older adults, urging increased physician awareness and patient referrals.

## Introduction

One in three adults, worldwide, faces a hidden threat called hypertension (HT). This chronic condition, where blood pressure (BP) is consistently elevated, silently raises the risk of cardiovascular diseases, kidney failure and dementia-related disorders. High systolic blood pressure, often without apparent symptoms, is estimated to claim 10.8 million lives per year.^1^

Hypertension insidiously wreaks havoc, compelling regular BP checks, medication adherence and lifestyle changes.^1^ Effective HT management is thus essential to reduce morbidity and mortality, especially for people 60 years and older.^2,3^ Unfortunately, older patients face challenges in controlling their BP (< 140/90 mmHg) because of factors like limited access to quality healthcare, poor adherence to recommended medications and difficulty in making the necessary lifestyle modifications.^4,5^ Illness perception, patient beliefs in the usefulness of the medication and illness burden play a significant role in treatment adherence patterns among older persons with HT.^5,6^

Amid these challenges, adult day care centres (ADCCs) emerge as a promising avenue for improving and managing HT. These community-based facilities provide care for older adults who live independently but require daytime supervision.^7^ Adult Day Care Centres offer varying services, including social activities, recreation, meals, essential health services and therapies. These activities promote independence, perceived physical health and social engagement and reduce the risk of institutionalisation.^8,9^

Regular physical activity and active social engagement are linked with decreased mortality, hypertension, cardiovascular disease, depression, falls and disability. Similarly, regular participation in social and productive activities and membership in large social networks have been shown to benefit health and functional outcomes as people age independently.^10^

Despite numerous ADCCs across South Africa, their impact on health outcomes, particularly BP control, remains largely unexplored. This study aimed to determine whether an effect was perceived on whether participation in ADCCs improves BP control and medication adherence among older adults in Bloemfontein, South Africa.

## Methods

### Study design and sampling

The study was initially designed to be a cohort study consisting of descriptive and analytical components. However, during data collection, the authors encountered a critical issue. The exposure group was unfortunately too small for meaningful comparison. This limitation rendered the analytical component infeasible.

The researchers will focus on the descriptive component of the study in this manuscript. This study was conducted at the outpatient department (OPD) of the Mangaung University of the Free State Community Partnership Programme Community Health Centre (MUCPP CHC) in the Mangaung Metropolitan Municipality of the Free State. Mangaung University of the Free State Community Partnership Programme (MUCPP) is a CHC situated in Phelindaba Township, in Mangaung, Bloemfontein. It is a health facility that serves mainly black people, the majority of whom are Sesotho-speaking.

The target population included all patients 60 years and older who have been receiving treatment for hypertension for a minimum of 2 years and receive their medication from MUCPP CHC. A convenient and purposeful sampling method was used to recruit at least 300 respondents. Patients who could not give informed consent because of intellectual or cognitive impairment were excluded.

### Data collection

From the study aim, a literature review was conducted, and a questionnaire was prepared by the researcher for respondents in their preferred language (English, Sesotho or Afrikaans). The study was conducted from July 2020 to September 2020. During this time, the primary researcher and research assistants conducted structured one-on-one interviews with the respondents using the questionnaire schedule and captured additional medical history from the clinic cards and patient files.

The information gathered included basic demographic information, socioeconomic information, respondents’ knowledge and attitude on hypertension and questions regarding the attendance or non-attendance of ADCCs.

The researchers limited disruption to the service delivery by interviewing only 15 patients daily. The primary researcher collected all completed questionnaires for safekeeping at the end of each day.

Because of the unforeseen limitation during participant selection and the project changing to a descriptive study, there were limits to the researchers’ ability to draw causal inferences. But, the questionnaire was initially tested for content validity through a comprehensive literature review, expert consultation and pilot testing. The unequal group sizes limited the generalisability of both validity and reliability estimates.

### Measurement errors and bias

The data may have been susceptible to recall bias, where respondents may have had inaccurate memories of past events related to their hypertension and ADCC participation.

### Pilot study

A pilot study was conducted on 10 patients from MUCPP CHC who met the inclusion criteria after receiving confirmed consent. Because of minor inconsistencies in the clarity of questions, some corrections were made to the questionnaire, and the results were excluded from the main study. The questionnaire was found to be valid for the intended purpose of the study.

### Data analysis

Following data collection, the information was transferred to a spreadsheet, securely maintained by the lead researcher. To ensure privacy, a data cleaning process was implemented to minimise the presence of identifiable information and to identify and correct data capturing errors. Data analysis was performed by the Department of Biostatistics, Faculty of Health Sciences, University of the Free State, using SAS version 9. Results of the descriptive variables were tabled in frequencies and percentages.

### Ethical considerations

The study received approval from the Health Sciences Research Ethics Committee of the University of the Free State (UFS-HSD2019/0606/0110) and the Free State Department of Health.

Before participation, all respondents were informed about the study and received an information letter in their language of choice. Respondents provided informed written consent. No patient was penalised or refused treatment for declining participation.

The researcher will keep all completed questionnaires safe and confidential for 5 years, after which the researcher will securely destroy them.

## Results and discussion

The original cohort study aimed to explore whether there is an association between ADCC attendance and effective HT management. Because of the error, we are reporting on the descriptive aspect of the study.

In total, 372 respondents met the inclusion criteria and consented to be included in the data analysis. Less than a quarter (23.4%) of the respondents have attended an ADCC. Most respondents were female, with a ratio of 3:1 (91.1% attending ADCC; 72.0% not attending ADCC), and 369 of the respondents were black African people and mostly Sesotho speaking while the remaining three were coloured people and Afrikaans speaking. The age median was 73 years for those attending and 68.5 years for those not attending. The age range spanned from 60 years to 95 years. Most respondents who attended ADCCs have attended for at least 4 years (48.9%), and 20% of respondents attended ADCCs for longer than 10 years.

Most respondents own the houses they live in (91.1%). The household arrangements ranged from 39 living alone, 86 living with a spouse, 192 living with their children and 224 respondents having grandchildren living in the household. The remaining small numbers live with parents, cousins or friends.

Of those attending ADCCs, many have some secondary level of education or more (51.1% attending, 30.9% not attending). Only a tiny number from both groups were still employed (2.2% of attending; 8.2% not attending), and the majority were retired and depended financially on old age grants (96.7% of attending; 96.1% not attending).

The clinical data show that more respondents attending ADCCs have controlled BP than those who have not attended (see Table 1).

**TABLE 1:** Attendance of adult day care centres compared to blood pressure control and reported medication adherence.

Variable	Attending (*n* = 90)		Not attending (*n* = 282)	
	*n*	%	*n*	%
**BP control**				
BP controlled†	64	71.1	145	51.4
BP uncontrolled	26	28.9	137	48.6
**Number of medications taken daily for HT**				
One	8	9.0	25	8.9
Two	31	34.8	105	37.5
Three	29	32.6	106	37.9
Four	19	21.4	32	11.4
Five	2	2.3	11	3.9
Seven	0	0	1	0.4
**Reported HT medication adherence (using as prescribed)**				
Yes	86	95.6	262	92.9
Sometimes	4	4.4	19	6.7
No	0	0	1	0.4

HT, hypertension; BP, blood pressure.

† , Controlled BP: < 140/90 mmHg.

The period since being diagnosed ranged from 2 years to 54 years. The median for those who attended ADCCs was 19.5, and for those not attending, it was 13.5. Most respondents from both groups perceived their hypertension as managed (67.8% for attending; 58.2% not attending), but a few were uncertain (7.8% for attending; 13.8% for not attending). Most respondents from both groups reported taking between 2 or 3 types of medication to manage their hypertension (see Table 1). Of those respondents attending, 95.6% reported taking medication as prescribed, compared to 92.9% from the group not attending. Most respondents took their medication independently (93.3% for attending; 93.3% for not attending), while those who answered ‘no’ stated that family members assisted them.

Only a tiny percentage of respondents reported ever stopping taking their medication (12.2% for attending; 8.9% for not attending). The reasons for stopping treatment included side effects, feeling healthy and missing follow-ups. When asked about other medical conditions, 29.8% of those who did not attend ADCCs complained about visual problems, while those who attended were 24.4%. Of those who attended, 22.2% were also aware of their kidney issues, while 23.3% previously had a cerebral vascular accident (CVA).

Of those not attending ADCCs, only 80 (28.4%) are aware of ADCCs in their area, and at least 21.6% will consider joining the ADCC if given the information on the ADCC benefits.

Most respondents who attended ADCCs did so for exercise (85.6%), to socialise (64.4%), to learn life skills (35.6%), for entertainment (11.1%) and a small number to develop healthy behaviour (7.8%). Other minority reasons accounted for 23.3%. Regarding the management of hypertension, respondents report that the ADCC offer them some assistance with regard to reminding them when to take medication (28.9%), when to go for follow-up (75.6%), lifestyle modifications like exercise (95.6%) and diet (90.0%), as well as health education (64.4%).

Challenges that are experienced by the respondents when they choose to attend the ADCC included health reasons (50.0%), family responsibilities (20.0%), distance (10.0%), affordability (10.0%), relocation (5.0%) and bad weather (5.0%).

Eighty-three respondents (92.2%) will recommend attending adult day care centres to other older people, with or without hypertension, given that it will benefit in the following manner: exercises, socialisation, entertainment, psycho-social support, assistance with family problems and life skills.

Recommendations that were given by some of respondents included the following: financial literacy, counselling sessions where necessary, self-development, donations of exercise equipment, sponsorships for touring, provision of dispensing machine at the ADCC for collection of chronic medication, delivery of chronic medication by the community health workers (CHWs) to ADCC, awareness campaign to inform other older persons about ADCC in their area, more financial and social support from the South African Social Security Agency (SASSA) and establishment of an investment fund to assist many older persons in times of need.

### Strengths and limitations

Only the descriptive aspect of the study is presented despite the survey being initially designed with an analytical component. Future studies should use an analytical component to confirm whether ADCC attendance is a positive influence.

## Conclusion

Despite this limitation, there seems to be a positive association between BP control among HT patients and patients attending ADCCs. No difference in treatment adherence could be shown. Still, those who attended ADCCs attest to the positive benefits of the ADCC, including reminding them to take the treatment, to go for follow-up visits and to assist with health education. This study indicates a strong positive influence from ADCCs, and further research with a robust design, such as a cohort study with a control group, is warranted to confirm these initial observations. It is a valuable tool for promoting health and well-being among this vulnerable population. Healthcare workers need to be made aware of the potential benefits of these facilities in their communities.
